# Maintaining wellbeing in remote work: a digital ethnography into resources, boundaries, and time

**DOI:** 10.3389/fpsyg.2026.1730559

**Published:** 2026-02-11

**Authors:** Serap Yalçınyiǧit

**Affiliations:** Department of Business Administration, Faculty of Economics and Administrative Sciences, Yıldız Technical University, Istanbul, Türkiye

**Keywords:** boundaries, conservation of resources, digital ethnography, remote work, subjective time, virtual mobility, wellbeing

## Abstract

**Aim:**

This study examines remote work as a form of virtual mobility and its implications for employees' subjective wellbeing (SWB). Using the complementary perspectives of Conservation of Resources (COR) and boundary theory, it presents a theoretical framework that explains how resource dynamics, boundary permeability, and time perceptions influence wellbeing in remote work.

**Methods:**

The study employs a digital ethnographic design based on 11 semi-structured interviews. These are complemented by field notes and an experiential elicitation task. The data were analyzed using reflexive thematic analysis, supported by computational techniques such as similarity measures, co-occurrence networks, and visualization tools implemented in R.

**Results:**

The findings identify three overarching themes: *temporal experiences*, in which acceleration, stagnation and fluctuating emotional rhythms that disrupted affective wellbeing; *blurred boundaries*, in which work-home permeability, erosion of collegial ties, and selective connections that reshape SWB; and *coping strategies*, which included boundary setting practices, job crafting, rituals, and recovery. The experimental task revealed a systematic bias toward underestimating time, consistent with participants' narratives of drift in monotonous work contexts.

**Conclusion:**

The study advances research into mobility and wellbeing theoretically by framing virtual mobility and multidimensional SWB, methodologically by showcasing the value of digital ethnography for capturing lived experiences, and practically by underlining the importance of organizational support and policies.

## 1 Introduction

The rapid adoption of digital work has transformed the way employees move, connect, and fulfill their roles, giving rise to a new type of mobility beyond physical mobility: *virtual mobility*. Traditionally, research has primarily focused on the physical aspects of mobility, such as migration, commuting, and studying abroad (Puura et al., 2017), often linking these movements to economic outcomes, including wages, occupational status, and career advancement (Fortunati and Taipale, 2017). While this body of work has offered valuable insights into the economic returns of mobility, it has paid far less attention to its implications for SWB (Müller et al., 2020). However, SWB is a fundamental aspect of human life and social progress (Diener, 1984), encompassing affective wellbeing (positive and negative emotions), social wellbeing (quality of relationships and sense of belonging), and cognitive wellbeing (life satisfaction and perceived balance) (Diener et al., 1999; Ryan and Deci, 2001; OECD, 2013). As remote work reshapes business dynamics, changing how employees shift between work and home spheres, manage multiple roles, and navigate digital environments, it influences their daily routines and wellbeing. Since this becomes a defining characteristic of contemporary organizations, it is both timely and necessary to understand virtual mobility and its consequences for SWB.

Recent societal transformations call for a rethinking of what constitutes mobility (Kaufmann, 2003). The rise of digital technologies and the expansion of remote working mean that individuals are increasingly engaging in virtual mobility. Digital integration has transformed the role of employees from passive execution of tasks to active, change-oriented contribution (Yalçınyiǧit and Karaçay, 2025). Meanwhile, virtual mobility has increased demands, as employees manage tasks and boundaries with less immediate supervision. Virtual mobility refers to movements that take place across various spaces, including organizational, interpersonal, and cognitive ones (Chudoba et al., 2005). These movements are made possible through the use of digital technology. This involves performing work and maintaining social relationships from a distance without relocating (Chudoba et al., 2005; Vartiainen, 2006). This requires crossing the boundaries between work and personal spaces while remaining spatially fixed, creating unique temporal and social conditions never experienced before (Sheller and Urry, 2006).

The study was approached through two complementary theoretical perspectives. COR (Hobfoll, 1989; Halbesleben et al., 2014) provides insights into how the resource dynamics inherent in telecommuting affect cognitive wellbeing. Gains such as autonomy, flexibility, and control over work rhythms can enhance satisfaction, while losses such as social isolation, blurred routines, and reduced access to support can disrupt the balance. Complementing this, boundary theory (Ashforth et al., 2000; Clark, 2000) illuminates how permeable work-life boundaries and the erosion of temporal markers affect emotional and social wellbeing as employees navigate these shifting boundaries. Together, COR and boundary theory provide an integrated framework for explaining how virtual mobility reshapes SWB through the interplay between resources, boundaries, and lived temporalities. Temporal experience serves as a conceptual bridge between COR and boundary theory. Within the COR framework, time is considered a psychological resource that individuals try to conserve, invest in, and protect (Hobfoll, 1989). Temporal exhaustion, such as fragmented attention or excessive cognitive effort, has been shown to contribute to strain by functioning as a form of resource loss (Beal et al., 2005). In contrast, boundary theory positions temporal rhythms as the structural and contextual conditions that regulate transitions between work and non-work domains (Ashforth et al., 2000; Clark, 2000). Remote work disrupts these rhythms by weakening the temporal markers that normally compartmentalize daily life (Wajcman, 2015). Consequently, employees experience time differently, a phenomenon that is becoming increasingly prevalent in digital work environments (Gregg, 2018).

Research on remote work is growing, but empirical evidence suggests that survey-based and cross-sectional designs, which capture broad relationships, overshadow qualitative research methods (Leonardi et al., 2024). Such approaches often overlook how employees experience time, emotions, and boundaries on-site and how these micro-level dynamics combine to create patterns of emotional, social, and cognitive wellbeing (Camfield et al., 2009; Inceoglu et al., 2018). There is limited qualitative evidence on how employees understand wellbeing in remote work, how resource cycles evolve in everyday digital interactions, and how temporal rhythms evolve when work and home life converge. To address this issue, the current study uses digital ethnography to offer detailed insights into the experiences of remote workers. By prioritizing workers' voices and everyday narratives, the approach advances methodological innovation in mobility and wellbeing studies and provides a contextual understanding of how virtual mobility is enacted and negotiated in practice.

Studies on remote work have largely focused on performance, productivity, or work-life balance (Leonardi et al., 2024). However, how employees experience time, emotions, and boundaries in relation to SWB remains to be investigated. As human behavior is driven not only by financial gain, but also by the quest for a fulfilling life, it is vital to explore the interplay between mobility and wellbeing to gain a more thorough grasp of mobility in modern societies. To this end, this study aims to achieve three interrelated objectives. First, by conceptualizing remote work as a form of virtual mobility, it examines how employees reconstruct their SWB through their lived experiences. Second, it integrates COR and boundary theory to develop a theoretical framework that demonstrates how resource dynamics and boundary permeability influence SWB in virtual mobility contexts. Third, it demonstrates the value of digital ethnography as a methodological approach for uncovering micro-processes of wellbeing not apparent in large-scale quantitative studies. These aims guided the research questions:

(1) How is SWB experienced and constructed in remote work?

(2) How do shifting temporal rhythms, work–home boundaries, and resource dynamics shape these wellbeing experiences?

(3) How do employees navigate the everyday transitions and tensions of remote work?

This study operationalized digital ethnography by combining semi-structured interviews, experiential tasks, and field notes. Through reflective thematic analysis, the research makes a psychological contribution by positioning subjective time perception as a key mechanism through which virtual mobility influences employees' affective, social, and cognitive wellbeing. By integrating COR and boundary perspectives through the lens of lived temporal experiences, the study shifts the focus from outcomes of remote work to the psychological processes through which employee wellbeing is constructed. Taken together, these contributions highlight virtual mobility as a distinctive condition of modern life and offer implications for both organizational design and public policy for SWB.

## 2 Theoretical framework

### 2.1 Virtual mobility

Mobility research has traditionally focused on physical movements (Cresswell, 2006). While this literature has illuminated how geographic relocation can generate opportunities and constraints, it has tended to treat mobility as merely the physical displacement of people across space (O'Carroll, 2014). Consequently, less scholarly attention has been devoted to forms of mobility that do not involve physical relocation. However, with the expansion of digital infrastructures and the normalization of remote work, mobility increasingly takes the form of virtual mobility, as the capacity to perform work, sustain relationships and move across organizational and role boundaries through digital means (Vartiainen, 2006; Selmer et al., 2022). Virtual mobility is not simply a functional substitute for physical co-presence (Urry, 2002). Rather, it generates a distinctive set of experiential and organizational conditions, with individuals enacting professional and personal roles in digitally mediated environments, negotiating boundaries across overlapping domains and experiencing the new temporal rhythms shaped by constant connectivity and flexible scheduling (Chudoba et al., 2005; Urry, 2007).

Unlike physical mobility, which primarily reshapes wellbeing through processes of displacement, resettlement, and integration, virtual mobility transforms the lived experience of time, affect, and social connection *in situ* (Erofeeva, 2025). While remote workers may not cross-national borders, they routinely traverse symbolic and relational boundaries between work and non-work spheres, creating novel demands for boundary management and resource allocation (Kreiner et al., 2009). Therefore, framing remote work as a form of virtual mobility highlights a distinctive mobility condition that warrants analytical attention (Vartiainen, 2006).

This conceptualization broadens mobility research by emphasizing the psychological consequences of non-physical movement, particularly how digitally mediated work reorganizes time perception, emotional regulation, and cognitive evaluations of wellbeing.

### 2.2 Resources, boundaries, and time

Remote work as a form of virtual mobility generates a complex interplay of resources (Hobfoll, 2001), boundaries (Katz, 1979; Ashforth et al., 2000), and experience of time (Buhusi and Meck, 2005; Thönes and Stocker, 2019) that shape employees' SWB. To capture these dynamics, we draw on COR and boundary theory, while also foregrounding the temporal dimension of work–life experiences. Together, these perspectives provide a comprehensive framework for understanding how virtual mobility reconfigures wellbeing conditions beyond physical movement in recent literature (Ren et al., 2024).

COR posits that individuals strive to obtain resources. They also strive to maintain and protect them. Stress emerges when these resources are threatened or depleted (Hobfoll, 1989, 2001). The creation of conditions of both resource gains and losses is a consequence of remote work (Stasiła-Sieradzka et al., 2023). Autonomy, flexibility, and control over work rhythms are offered to employees, with the potential to enhance life satisfaction and perceived balance (Ren et al., 2024). Theoretically, if we assume that work life is organized around home and workplace location, and the two are linked by commuting distance, commuting can be viewed as a cost (Rüger et al., 2024). However, remote work can also lead to losses, such as reduced social support, disrupted routines, and unclear role expectations, which can undermine vitality and job satisfaction (Costin et al., 2023). These dynamics demonstrate that cognitive wellbeing is not solely determined by mobility, but by the cycles of resource accumulation and depletion triggered by virtual mobility. It is important to note that resources are interdependent and that gaining temporal flexibility may result in a loss of collegial support and have mixed effects on wellbeing.

While COR explains resource-based processes, boundary theory sheds light on the influence of permeable work–life borders on social and affective wellbeing (Ashforth et al., 2000). Remote work dissolves the traditional spatial and temporal markers of work, making it difficult for employees to distinguish between their professional and personal roles (Hardill and Green, 2003). These blurred boundaries increase the permeability between domains, resulting in a sense of temporal drift where work and personal time merge without clear transitions. These conditions affect SWB by weakening collegial interaction and altering family dynamics, as the absence of clear boundaries reshapes relational practices (Song and Gong, 2025). They also impact affective wellbeing by disrupting emotional rhythms, meaning that, without structured boundaries, employees oscillate between stress, monotony and moments of vitality (Johnson and Mabry, 2022). Therefore, boundary permeability is neither inherently positive nor negative; its effects depend on how individuals navigate the intersection of roles, and on the extent to which boundaries enable or constrain relational and emotional regulation.

Beyond resources and boundaries, virtual mobility is distinctive in the way it reconfigures temporalities. Temporalities refer to how individuals perceive, structure, and live through time in everyday practices (Adam, 1995; Wajcman, 2015). Conceptions of time have long oscillated between seeing it as an independent reality, Platonism, or as contingent on worldly events, reductionism (Fischer, 2016). Thinkers such as Aristotle emphasized time as a measure of change (Coope, 2005), while Augustine and Kant located it in the human mind (Chojnacki and Żardecka, 2018). Contemporary neuroscience complements these views by showing how the brain encodes temporal information through sensory patterns (Eagleman et al., 2005). While temporal rhythms had long been shifting, the global pandemic accelerated and intensified these changes, further altering how time is perceived, structured, and inhabited (Loose et al., 2021). In remote work, employees report accelerated workdays, slowed or monotonous periods, and blurred transitions between beginnings and endings (Altaf et al., 2023). Such temporal drift has profound implications for SWB: accelerated rhythms may intensify stress and emotional exhaustion, while monotonous rhythms may erode engagement and vitality (Velleman, 1991). At the same time, flexible temporalities allow for job crafting and self-regulation, thereby enhancing autonomy and satisfaction (Wessels et al., 2019). We emphasize how virtual mobility reorganizes social and resource structures and reshapes the lived experience of time. This is a critical dimension for understanding wellbeing in mobility contexts.

Resources, boundaries, and temporalities function as interconnected mechanisms through which virtual mobility shapes wellbeing. This framework highlights their interdependence and situates them within virtual mobility, advancing a multidimensional view of how non-physical mobility reshapes SWB.

### 2.3 Multidimensional view of SWB

Traditional approaches have predominantly defined SWB as comprising affective states (positive and negative emotions) and cognitive evaluations of life satisfaction (Diener et al., 1999). Building on these foundations, scholars have emphasized that social relationships are a critical yet often underexamined dimension of wellbeing, coining the concept of social wellbeing to capture feelings of belonging, contribution, and connectedness (OECD, 2013; Helliwell et al., 2025). Remote work as a form of virtual mobility provides a particularly compelling context in which to foreground all three dimensions simultaneously, as it reorganizes emotional rhythms, relational practices, and cognitive evaluations of balance and satisfaction.

Firstly, the blurring of work–life boundaries and the drift of temporal markers directly shape employees' emotional lives (Anderson et al., 2015). Experiences of stress, monotony, and vitality are tied to how individuals regulate affect under conditions of temporal acceleration or stagnation (Zhao and Yuan, 2025). Boundary theory helps explain how the erosion of transitions between work and personal time destabilizes affective rhythms, while COR highlights how resource loss cycles (e.g., reduced social support) amplify negative emotions (Westman et al., 2004). Second, remote work alters the quality and availability of social relationships. Reduced collegial contact and the loss of informal interactions can undermine a sense of belonging and integration (Golden et al., 2008). Research shows that the absence of spontaneous encounters in remote work often leads to professional isolation, weaker organizational identification, and diminished opportunities for social learning (Bartel et al., 2012). Meanwhile, intensified family proximity may either strengthen or put a strain on household ties (Allen et al., 2014). While constant presence can foster closeness and shared routines, it may also increase conflict and role overload, especially when boundaries between work and family blur (Allen et al., 2014). Social wellbeing thus emerges as a critical outcome of virtual mobility, shaped by the permeability of boundaries and the distribution of social resources. Finally, employees assess their work and life satisfaction considering resource dynamics (Heller et al., 2006). Gains such as autonomy and flexibility support a sense of control and accomplishment, while losses such as blurred routines or role ambiguity detract from perceived balance (Deci and Ryan, 1987). COR Theory clarifies how these cycles of gain and depletion underpin cognitive evaluations of wellbeing (Baer et al., 2018).

## 3 Materials and methods

### 3.1 Research design

This study uses a digital ethnographic approach to examine how employees experience and narrate remote work as a form of virtual mobility, and how these lived processes influence SWB. Digital ethnography is well-suited to this line of enquiry as it highlights *in situ* practices, temporal rhythms and modes of communication mediated by digital infrastructures (Hine, 2015; Pink et al., 2016). While survey-based and cross-sectional approaches capture broad associations between remote work and wellbeing, they often overlook the micro-processes through which boundary permeability, resource dynamics and temporal experiences unfold in everyday routines (Camfield et al., 2009). By prioritizing participants' own accounts and contextual cues, digital ethnography enables us to trace how meaning, emotion and time are co-constituted in digitally mediated workspaces (Cleland and MacLeod, 2022).

### 3.2 Participants and data collection

Data were generated from 11 full-time remote workers who were recruited using purposive and snowball sampling methods (Patton, 2015). The semi-structured interview form was developed for this study. An English version of the interview guide is provided as supplementary material. With the pandemic, mobility emerges as a space that reveals inequalities as well as access to equality. In fact, viewing mobility as an opportunity or a vulnerability is tied to individual experiences (Almeida et al., 2025). Therefore, we included participants from diverse backgrounds in the sample to capture diverse lived experiences. The sample included both men and women with an age range of 26 to 39 years, and represented a variety of occupational sectors, including business services, education, the creative industries, and technology. Participants have 1 to 4 years of remote working experience, with a focus on those who established routines. These contextual details demonstrate how different demographic and organizational conditions influence employees' temporal rhythms, boundary experiences, and wellbeing in remote work settings, thereby supporting the transferability of findings. Further information can be found in Table 1.

**Table 1:** Participants.

**Pseudonym**	**Age**	**Background**
Jack	28	Mechanical engineer, working in the renewable energy sector; holding an MBA; married, no children. Typically participating in 1-2 online meetings per week.
Marcus	27	Patent officer; bachelor's degree; single, no children. Attending daily online meetings.
Emily	26	Researcher at a state university; master's degree; single, no children. Usually joining one online meeting per month.
Larry	30	Compensation and reporting manager; master's degree; single, no children. Usually joining two online meetings per month.
Julia	29	System engineer; bachelor's degree; married, no children. Attending daily online meetings.
David	27	Intellectual property officer; master's degree; single, no children. Attending daily online meetings.
Jessia	35	Financial manager; bachelor's degree; single, no children. Usually joining 1-2 online meetings per week.
Ruby	33	Researcher; PhD; single, no children. Usually joining one online meeting per week.
Mia	32	Product manager; master's degree; married, no children. Attending daily stand-up meetings and weekly planning sessions.
Austin	39	University faculty member; PhD; married, two children (primary school age). Usually attending 2-3 online meetings per week, most of which were committee meetings.
Eva	28	Software developer; master's degree in computer engineering; single, no children. Usually attending 2-3 short stand-up meetings per day.

Participants lived in various household configurations (alone, with partners, or with families), enabling the examination of wellbeing under different social and organizational conditions. Participants were recruited via professional networks and personal contacts, with invitations sent via email and messaging platforms. All participants provided informed consent, and pseudonyms (e.g., Jack, Emily, Larry, and Julia) are used throughout to preserve anonymity.

In qualitative research, sample size is determined based on the principle of saturation rather than statistical generalizability. Saturation occurs when no new themes or insights emerge from the data (Guest et al., 2006). Previous research indicates that thematic saturation in interview-based studies is typically achieved with 6–15 participants (Braun and Clarke, 2006). The study reached thematic saturation with 11 participants. The first seven to eight interviews generated all the higher-level themes (blurred boundaries, temporal experiences, and coping strategies), and subsequent interviews produced only minor variations (Saunders et al., 2018). The coding memos and reflexive notes confirmed that saturation had been reached because the final interviews reinforced patterns that had already been identified. Thus, saturation was determined by both sample size and repetition of analytic categories across participants. Furthermore, the sample was diverse in terms of sector, job position, gender, and family status, increasing the breadth of perspectives captured. The use of multiple data sources, including observations and a supplementary field observation in addition to interviews, further enhanced the richness and reliability of the findings, supporting the adequacy of the study.

*Semi-structured interviews:* These were conducted online via Zoom and lasted 35–55 mins. Although the interview guide focused on structured topics, temporal experiences of remote work, affective states, work-life boundaries, participants were encouraged to elaborate freely, and any emerging issues were explored. Questions such as “How does your mood compare now to when you started working from home?” and “Do you feel there are differences in your perception of time during the day or in certain areas of your life?” were used alongside targeted probes to elicit specific examples of daily routines and emotions. The semi-structured format ensured comparability across participants, allowing them to shape the conversation, highlight personally relevant issues and expand on emerging themes.

*Experiential elicitation task:* Elicitation tasks are commonly used in qualitative research to assist participants in expressing cognitive and emotional experiences that are challenging to articulate verbally (Bagnoli, 2009). Thus, this study designed a task to capture momentary temporal judgments, providing an experiential anchor to enhance participants' reflections. Participants watched a 30 s video filmed in a workplace meeting room. In the video, individuals performed repetitive actions such as tapping pens, flipping through documents, and adjusting laptops. This sequence was deliberately monotonous, designed to encourage participants to experience temporal drift and estimate its duration. All participants viewed the video once, under the same standardized procedure. Before the task, participants were instructed to watch the clip without interruption and estimate its duration immediately afterward. The video could not be paused or replayed, ensuring consistency across participants. This exercise prompted reflections on the perception of time and revealed systematic biases, complementing the interview narratives.

*Contextual field notes and reflexive observation:* Notes were taken during and after each session. These notes captured participants' digital routines (e.g., daily calendar, meeting rhythms) as well as non-verbal cues such as tone, pauses, and affective shifts. Reflexive observations documented how the digital medium shaped interactions (e.g., background settings, connectivity issues). Together, these accounts enriched the dataset by adding contextual depth beyond verbal content.

All interviews were audio-recorded with permission and transcribed verbatim. These transcripts were then enriched with observational notes to preserve contextual and emotional nuances. Together, these materials formed a multimodal corpus that anchored the analysis.

### 3.3 Analytical strategy

First, the transcripts were read repeatedly to achieve immersion, with preliminary notes being made on salient expressions relating to time perception, emotions, boundaries and resources. The data were analyzed using reflexive thematic analysis, following the guidelines of Braun and Clarke (2006). All transcripts were read line by line to generate initial open codes that captured semantic content and emerging latent meanings. These codes were gradually refined into more specific ones through constant comparisons across interviews (e.g., “*affective ups and downs over the day,” “blended home-work routines,” “weakened collegial bonds,” “tailoring job to self,” “time feels fast”*). The codes were then grouped conceptually to create preliminary thematic structures. These structures were revisited and reshaped as new connections emerged.

Themes were validated through an iterative process aimed at ensuring internal consistency and analytical robustness. Within each theme, codes were checked for internal homogeneity, and themes were compared against each other to ensure external heterogeneity (Patton, 2015). The final themes were identified after a repeated review of the coded quotes, notes, and analytical diagrams was conducted to ensure a strong connection to the participants' life experiences. The emerging structure was interpreted through the study's sensitizing concepts of resources, boundaries, and temporalities, which were mapped onto the affective, social, and cognitive dimensions of SWB via reflexive thematic analysis (Braun and Clarke, 2006, 2021).

**Figure 1:**
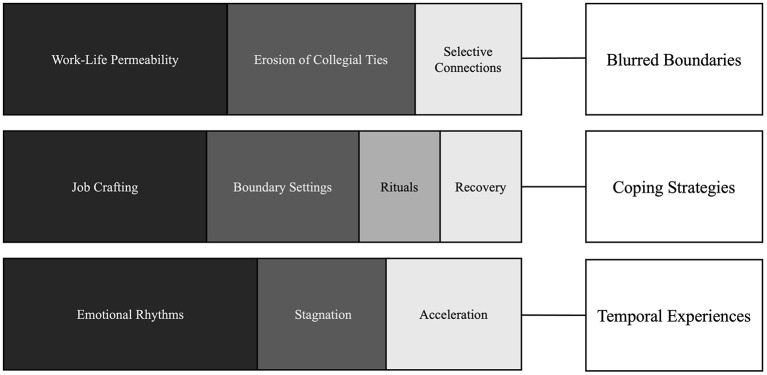
Themes and subthemes.

To strengthen the analysis and enhance transparency, R was used to generate descriptive outputs such as co-occurrence networks, similarity tests and heat maps. To avoid including incidental links, the co-occurrence threshold was set at a minimum of two occurrences within the same meaning unit. Visualizations were generated to support reflexive thematic analysis by showing relational patterns among codes. Clusters in the networks and heat maps were qualitatively interpreted to confirm themes that emerged regarding boundaries, temporal rhythms, and resource dynamics. These visualizations were not used for statistical generalization, but rather as tools for making sense of the data that helped to identify relational patterns and thematic intensities. Finally, the themes were reviewed, defined and refined, resulting in three overarching categories, as blurred boundaries, coping strategies, and temporal experiences. In Figure 1, top subthemes by theme are illustrated.

Reflexivity was at the center of the analysis. Throughout data collection and analysis, the researcher kept reflexive memos to identify and limit possible biases. These memos documented moments when the researcher's experiences might have influenced interpretation, and they were reviewed during the coding process. Leading statements were avoided during semi-structured interviews. The researcher's familiarity with academic and hybrid settings provided contextual insight but also required conscious reflection to avoid over-identifying with the participants. These reflexive practices increased the transparency and reliability of the analytical process. Before reporting, in line with best practices for qualitative research, participants were also given the option to review selected excerpts of their interviews prior to publication, ensuring that their narratives were represented fairly and respectfully.

## 4 Results

The analysis generated a set of interrelated themes that capture the related research questions. Before delving deeper into the relationship between each theme and SWB, these visualized results are reported to enhance clarity and provide a comprehensive overview.

Table 2 shows how the emergent sub-themes are distributed among the participants. A total of 455 coded segments were identified, reflecting a rich and varied set of experiences. The most frequently observed sub-themes were emotional rhythms (*n* = 72), job crafting (*n* = 68) and work–life permeability (*n* = 61). This suggests that fluctuations in emotional states, individual adaptation strategies and the permeability of work–life boundaries were central aspects of participants' remote work narratives. Mid-range themes such as boundary setting (*n* = 51) and erosion of collegial ties (*n* = 51) highlight the tension between efforts to establish structure and the challenges of diminished collegial contact. Less frequently reported, yet still analytically significant, sub-themes included stagnation (*n* = 37), acceleration (*n* = 36), rituals (*n* = 29), selective connections (*n* = 27) and recovery (*n* = 23). Although these were mentioned less frequently, they provide valuable insights into how employees navigated temporal drift, everyday coping strategies, and selective forms of relational engagement. At the participant level, some narratives were particularly extensive (e.g., Jack with 84 coded segments and Larry with 62), while others were more concise yet focused (e.g., Julia with 30 and Eva with 29). Notably, job crafting emerged strongly in Larry's and Jessia's accounts, whereas emotional rhythms were especially salient in Jack's and Emily's narratives. These variations demonstrate that, although overarching themes cut across cases, the intensity and configuration of sub-themes differ, highlighting the heterogeneity of SWB under conditions of virtual mobility.

**Table 2:** Emergent sub-themes based on participants' experiences.

**Sub-theme**	**Jack**	**Marcus**	**Emily**	**Larry**	**Julia**	**David**	**Jessia**	**Ruby**	**Mia**	**Austin**	**Eva**	**Sum**
Work-life permeability	14	5	4	9	3	4	5	5	1	5	6	61
Erosion of collegial ties	11	2	5	10	4	5	-	3	3	4	4	51
Selective connections	8	2	-	3	-	1	4	1	3	4	1	27
Boundary setting	5	1	5	9	6	5	9	2	2	2	5	51
Job crafting	6	7	6	14	3	5	10	7	8	-	2	68
Rituals	4	1	4	2	1	3	-	1	8	3	2	29
Recovery	2	-	6	4	-	4	-	3	2	1	1	23
Acceleration	6	5	2	1	4	2	5	5	2	2	2	36
Stagnation	12	4	4	3	2	3	1	2	1	2	3	37
Emotional rhythms	16	7	9	7	7	7	8	3	3	2	3	72
Sum	84	34	45	62	30	39	42	32	33	25	29	455

**Figure 2:**
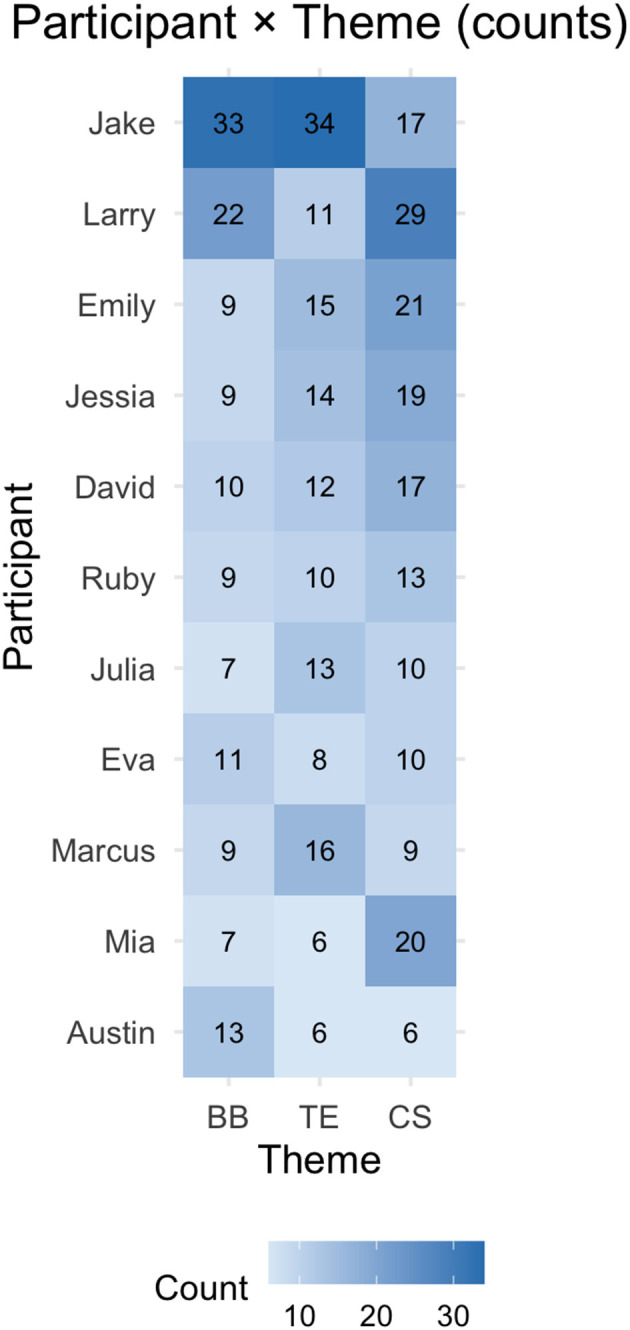
Distribution of themes across participants (counts).

**Figure 3:**
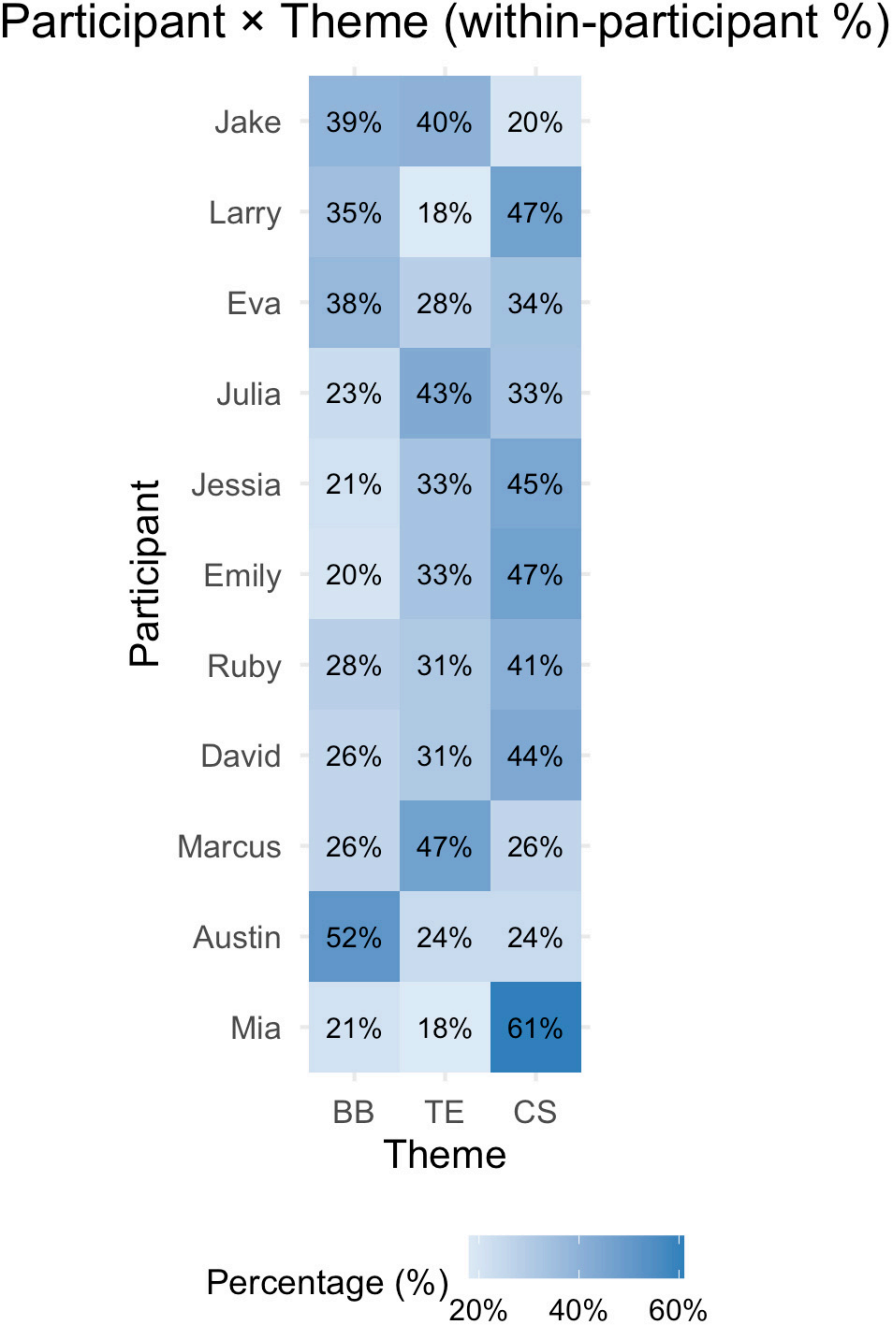
Distribution of themes across participants (within-participant %).

Figures 2, 3 show how the three overarching themes are distributed among the participants, the heatmaps, in both raw counts and within-participant proportions.

The count matrix shows that blurred boundaries and temporal experiences were the themes that were discussed most consistently, particularly in the accounts of Jack (blurred boundaries: *n* = 3; temporal experiences: *n* = 34) and Larry (blurred boundaries: *n* = 22; temporal experiences: *n* = 11). In contrast, coping strategies were mentioned less frequently overall, though they still featured strongly in certain participants' narratives (e.g., Jack, Larry, and Austin). When normalized as percentages, clear variations emerge in how participants weighted the themes within their own accounts. For example, coping strategies dominated Austin's narrative (63%), whereas experiences of temporal experiences outweighed other themes in Jack's narrative (48%). Ruby and Eva, on the other hand, provided more balanced accounts, with blurred boundaries, temporal experiences and coping strategies comprising around 30–35% of their coded segments each. These proportional differences not only highlight which themes were most salient across the dataset but also demonstrate how individuals varied in the emphasis they placed on boundaries, time, or coping. This heterogeneity emphasizes the various ways in which remote workers navigated virtual mobility and its implications for SWB.

**Figure 4:**
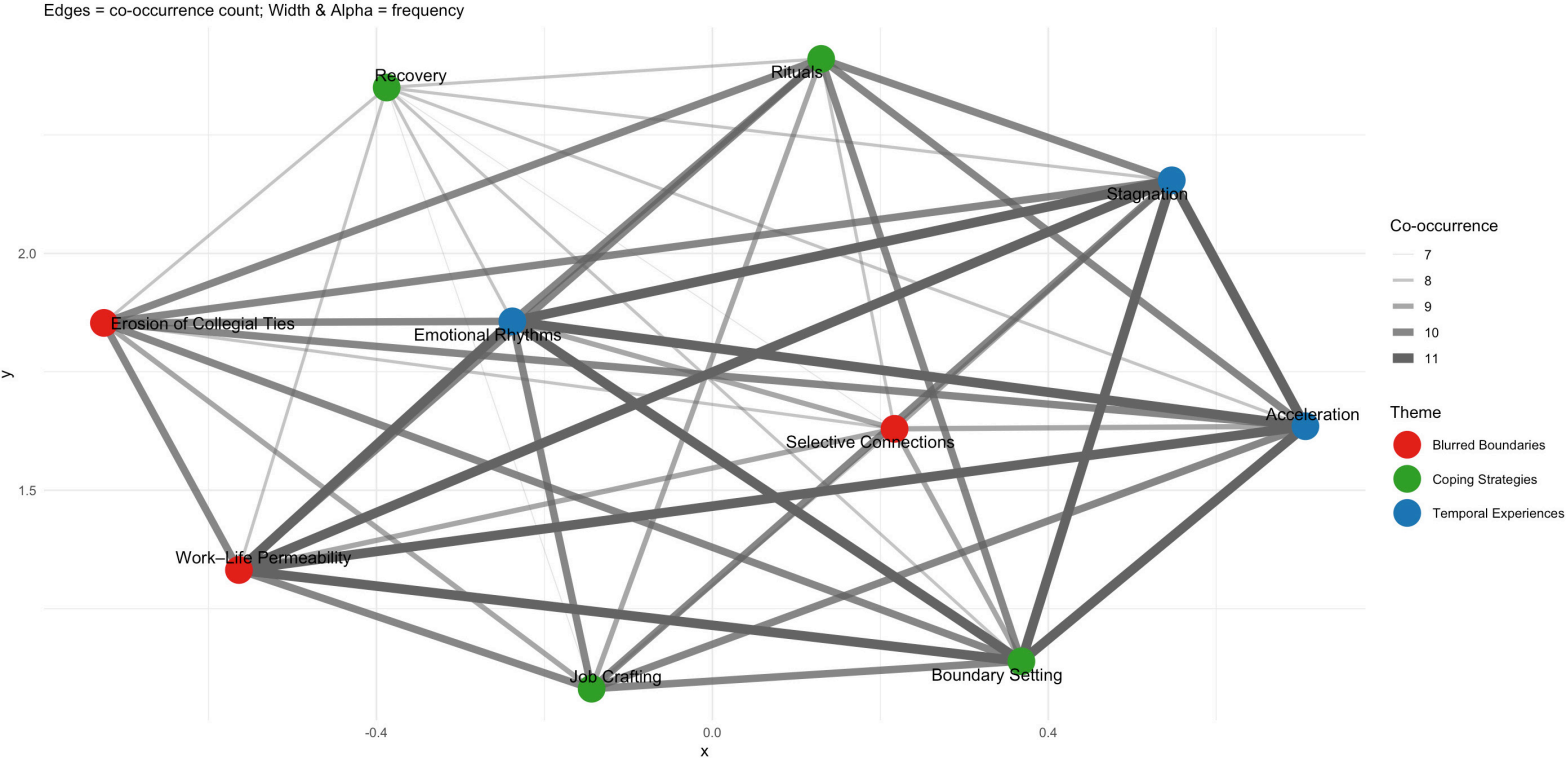
Co-occurrence network of sub-themes.

In addition, Figure 4 illustrates the co-occurrence network of subthemes, where edge thickness reflects the frequency of co-occurrence across participants' narratives.

The visualization shows that work–life permeability (red, blurred boundaries) and emotional rhythms (blue, temporal experiences) acted as central nodes, forming strong connections with a wide range of other subthemes. For instance, work–life permeability frequently co-occurred with boundary setting and job crafting, suggesting that participants often discussed the merging of work and home boundaries alongside strategies to re-establish separation or regain control. Similarly, emotional rhythms were closely tied to both “recovery” and “stagnation”, reflecting how fluctuations in mood were embedded in the temporal experiences of acceleration, slowing down or regaining energy. The network also reveals cross-domain linkages, showing that coping-related subthemes (e.g., recovery and job crafting) were closely intertwined with boundary and temporal themes. This suggests that participants' adaptive strategies were not isolated practices, but rather interconnected responses to B blurred boundaries and shifting temporalities. The density of the network highlights the interdependence of resources, boundaries and temporal dynamics in shaping SWB in the context of virtual mobility.

**Figure 5:**
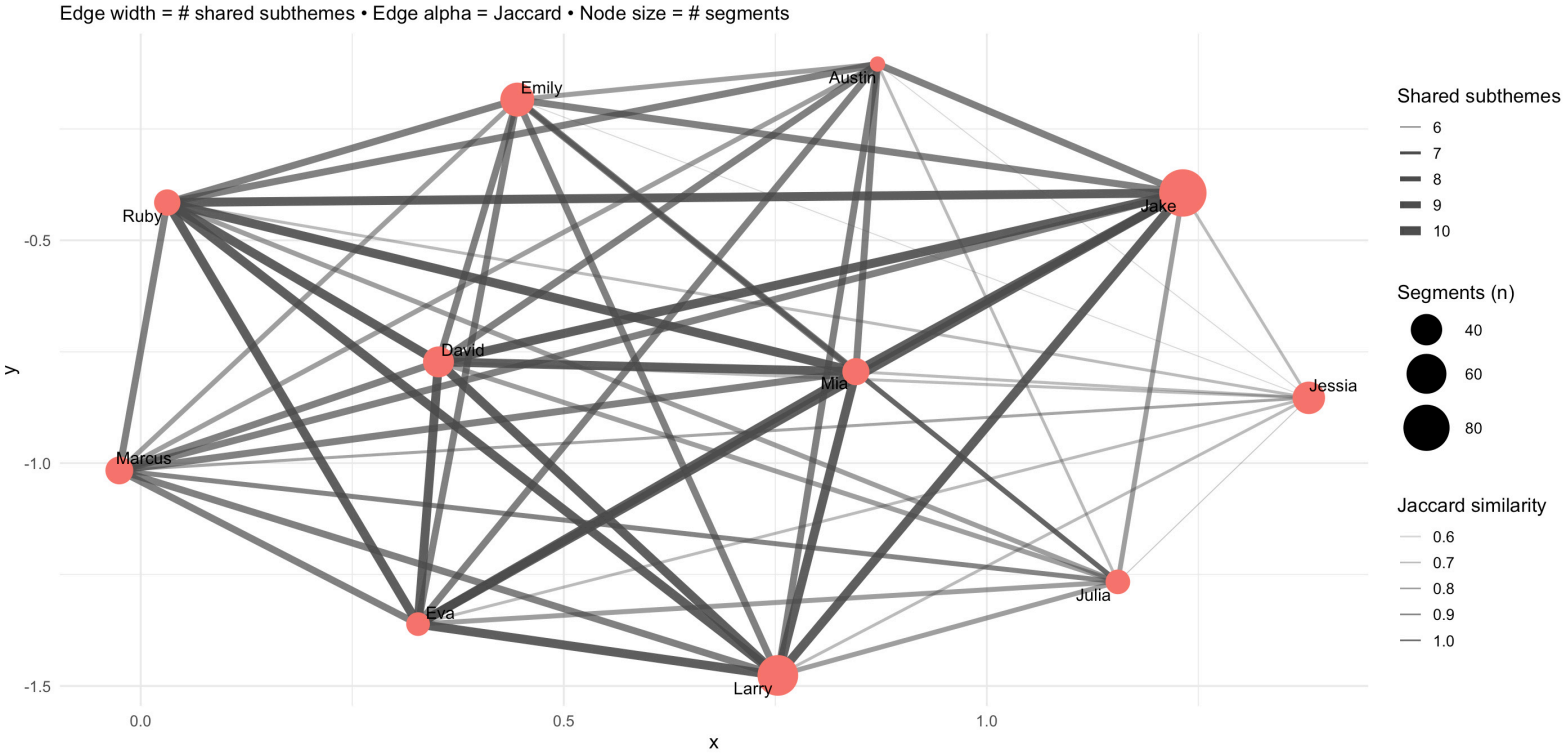
Participant social network via shared subthemes.

Lastly, Figure 5 visualizes the degree of thematic similarity among respondents in the form of a participant social network, based on the number of shared subthemes across their narratives. Node size reflects the number of subthemes expressed by each participant, while edge thickness and opacity indicate the strength of overlap (Jaccard similarity). The clustering of ties shows that participants widely shared temporal experiences and blurred boundaries, creating dense interconnections. By contrast, coping strategies appeared more unevenly distributed, with practices such as job crafting or micro-rituals concentrated among a subset of participants. This pattern suggests that, although the erosion of collegial ties and emotional rhythms was widely recognized, participants' responses to these challenges diverged, resulting in variation in coping strategies. Taken together, the network reveals both the collective dimension of remote working experiences, particularly regarding boundary permeability and temporal disruption, and individual variation in adaptive strategies. These findings demonstrate how shared conditions of virtual mobility can generate both overlapping and heterogeneous trajectories of SWB.

**Figure 6:**
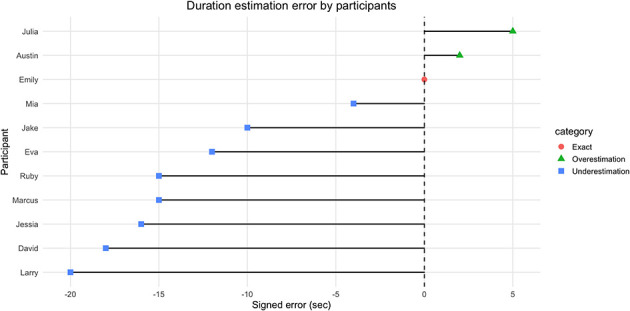
Duration estimation error by participants.

Additionally, participants were asked to estimate the duration of a repetitive 30 s video. The results revealed a systematic underestimation bias: on average, participants underestimated by 9.36 seconds (SD = 8.69), with a median error of −12 s and a mean absolute error of 10.64 s (MAPE = 35.5%). The majority (73%) underestimated the duration, while only two participants (18%) overestimated it, and one participant provided the exact estimate. A Wilcoxon signed-rank test confirmed that the median error significantly differed from zero [V = 4, *p* = 0.019, 95% CI (−16.5, −4.0)]. Figure 6 shows that Larry and David were the most extreme under estimators, with errors of −20 s and −18 s respectively, while Austin and Julia were the most extreme over estimators, with errors of +2 s and +5 s respectively.

The lollipop chart shows individual-level estimation errors relative to the true duration of 30 s. A clear pattern of underestimation emerged, with most participants misjudging the video as shorter than it was. Larry and David represented the most extreme underestimations, with errors of −20 s and −18 s respectively, followed by Jessica and Marcus with errors of around −15 s. Only two participants, Austin (+2 s) and Julia (+5 s), overestimated the duration, while Emily provided an exact estimate. This pattern highlights a consistent bias toward perceiving time as compressed in remote working contexts. The predominance of underestimation aligns with participants' narratives of temporal acceleration, suggesting that the subjective experience of “days passing too quickly” is reflected in measurable distortions of time perception. At the same time, the few cases of overestimation highlight the heterogeneity of experiences, particularly in contexts marked by monotony or low engagement.

Having presented the distribution of subthemes among participants and the interrelations among themes and individuals within the network, the analysis will now turn to a more detailed exploration of the three overarching thematic domains.

## 5 Discussion

**Figure 7:**
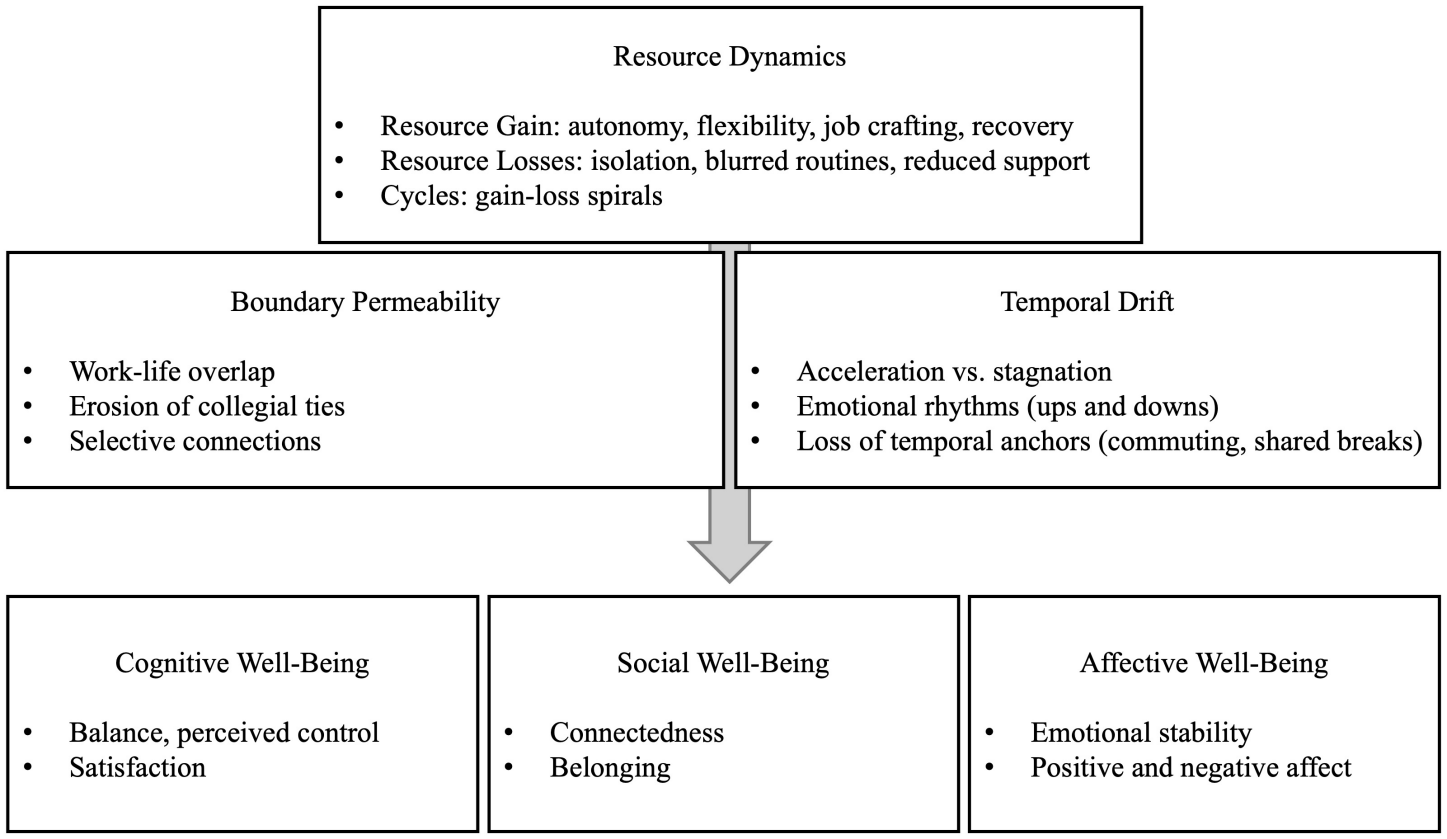
Conceptual model.

This study advances organizational psychology by demonstrating that the effects of remote work on wellbeing are not merely structural or contextual, but fundamentally psychological. Specifically, subjective time perception emerges as a core mechanism through which resource dynamics and boundary permeability are translated into affective, social, and cognitive wellbeing outcomes. The conceptual model, as shown in Figure 7, developed in this study explains the effects of remote work on SWB at a process level by revealing the interactions between resource dynamics (COR), boundary theory, and temporal affects.

The three core components in the top row of the model represent different dimensions of virtual mobility:

- The gain of resources such as autonomy, flexibility, and control in remote work, and the loss of resources such as lack of social support, uncertain routines, and increased loneliness.
- The transformation of employees' emotional rhythms and social ties due to permeable boundaries between work and life spaces. This process encompasses both the erosion of professional
- Cycles of acceleration, deceleration, or stagnation of time resulting from the loss of physical markers.

These three elements are interconnected in the model as a process. Cycles of resource gain/loss, boundary permeability, and the shifting of temporalities interact with each other. However, each component also has a direct impact on SWB outcomes. This framework addresses SWB not only in a results-focused but also process-focused way, providing a holistic perspective on how employees manage their resources, negotiate their boundaries, and experience time.

### 5.1 Temporal experiences: affective wellbeing

Participants consistently emphasized how remote working altered their perception of time, affecting their emotional state and sense of rhythm (Rosa, 2013; Thönes and Stocker, 2019). Temporal experiences emerged as a key aspect of affective wellbeing, with accounts oscillating between feelings of acceleration, stagnation and fluctuating emotional rhythms.

For many, remote working intensified the pace of daily life. Meetings, tasks and digital communications seemed to merge into what felt like shorter workdays.

“I was so distracted at the office, wondering where to start... Time passed slower when I was in the office, but now it's much faster. There's a fivefold difference....My perception of time has changed. A moment ago it was 11 a.m. Now it's 3:30 p.m. It scares me how fast time goes by, it feels like I haven't done anything, and it's just flown by.” (Ruby)

“After working from home, my days started to look so like each other that I lost track of months. This made time fly by so quickly.” (Eva)

Emily echoed this sense of compression:

“Days are passing by so quickly, it's as if I start in the morning and before I know it, it's evening....When I'm at work, I have to start an experiment very early because there can be a 6 h wait. I start the experiment at 6 a.m. so I can finish it early and get home early. I finish all my work before the experiment ends, but then I realize it's only 1 a.m.”

The absence of commuting and in-office breaks eliminated conventional temporal markers, creating a condensed perception of time (Rosa, 2013). While acceleration sometimes fostered productivity, it also generated strain and emotional exhaustion (Xanthopoulou et al., 2012). In contrast, several participants described time as slowed or stagnated. Mia observed:

“Time speeds up in deep focus sessions; in operational work, it slows down when the flow is interrupted. I have a window, especially between 1:30 PM and 3:30 PM, where time stops.”

“Some days feel endless, hours drag on.” (Larry)

Without spatial or social transitions, days blended into one another, creating monotony and emotional heaviness. Previous studies show that informal overtime at home is associated with increased mental health complaints, whereas remote working methods are linked to fewer mental and physical health complaints, especially at lower levels (Mergener et al., 2025). Temporal drift was associated with frustration, low mood and reduced engagement (Flaherty, 1999).

Participants also emphasized how temporal shifts are directly intertwined with emotional rhythms.

“When the workday ends, I don't even change where I sit. …When I'm engrossed in work, time disappears, but when I finish, I feel incredibly tired.” (Marcus)

“When I forget to take a break, I feel irritable and exhausted.” (Julia)

These reflections illustrate moments of flow during immersion, which are often followed by abrupt drops in energy and mood once interrupted. Emotional rhythms thus represent not only reactions to temporal perception, but also key mechanisms that shape affective wellbeing (Sonnentag, 2015).

Taken together, these findings demonstrate how virtual mobility can destabilize temporal anchors, such as start and end times, commuting routines and shared breaks, leading to volatile emotional states (Mazmanian et al., 2013). While acceleration produced productivity, it also produced strain, and deceleration and drift produced monotony and fatigue (Flaherty, 1999; Xanthopoulou et al., 2012; Rosa, 2013). Systematic underestimation of duration reinforces participants' narratives of temporal acceleration and drift, emphasizing the importance of temporal experiences in shaping SWB.

### 5.2 Blurred boundaries: social wellbeing

Remote work disrupted participants' experience of time and reshaped the boundaries between professional and personal life. These blurred boundaries had a significant impact on SWB, resulting in both strain and selective forms of connection. Three interrelated subthemes emerged: work–home permeability; erosion of collegial ties; and selective connections.

Participants often described how professional and domestic responsibilities merged in ways that undermined clear separation, which appeared as work-home permeability.

“When you work from home, you don't feel like you're working. Your routine changes, and your personal life becomes chaotic.” (Jessia)

Austin echoed this, noting that he frequently found himself multitasking:

“The main issue for me was the mixing of work and family time. …I'm in a meeting, and I'm also looking after the kids. The two things are getting mixed up. …my fatherly role is always active at home.”

Empirical evidence regarding individuals' quality of life suggests that working conditions are particularly important for individuals' overall life satisfaction (Kurowska et al., 2025). Similarly, while some participants appreciated flexibility, most expressed the stress, focus dilation, and sense of presence associated with permeability. In previous studies, flextime working arrangements, where employees can determine their own start and end times, were found advantageous in terms of maintaining home-work boundaries (Lott, 2020).

Another recurring challenge was the erosion of collegial ties. Several participants missed the spontaneity of interactions and the informal support of office life.

“You miss your friends... there were times when I wished I was there.” (Jessia)

“…even though I'm not in the office right now, I still miss going out for lunch. Nothing can replace the conversations we used to have during those lunches or coffee breaks at the office.” (Larry)

These accounts illustrate how virtual mobility reduces opportunities for casual encounters, which are crucial for a sense of belonging and social wellbeing. However, participants also acknowledged that digital platforms partially filled this gap.

“Video calls are helpful to a certain extent, but they're not the same as face-to-face.” (David)

Previous studies show that although virtual working arrangements due to high independence, larger flexibility, reduction in commute time, it can include complexity and uncertainty in communication that affects even the workplace isolation behavior among employees (Munir et al., 2016).

Interestingly, some participants deliberately limited their social engagement, using blurred boundaries as an opportunity for selective connection.

“I don't miss the work environment... I just miss my friends and our conversations.” (Ruby)

“I miss the work environment. After all, I miss the social environment at work. When you had a break there, going downstairs for 10 mins or going out to the terrace for a coffee were fun activities.” (Marcus)

These narratives highlight how virtual mobility can enable employees to redefine their social priorities, allowing them to shed superficial ties while preserving meaningful ones. However, this selectivity was often viewed ambivalently. While it provided an escape from unwanted social interactions, there was a risk that it would reduce overall social support.

Thus, the blurring of boundaries between work and personal life produced a paradox for SWB. On the one hand, permeability and isolation eroded participants' sense of balance and belonging (Ashforth et al., 2000; Mazmanian et al., 2013). On the other hand, selective connection enabled some to prioritize and strengthen chosen relationships (Golden et al., 2008). Together, these dynamics emphasize that virtual mobility reshapes the social fabric of work, simultaneously limiting and altering opportunities for social integration.

### 5.3 Coping strategies: cognitive wellbeing

Participants developed a range of coping strategies to help them regain a sense of control, balance and satisfaction while working remotely. These strategies centered on setting boundaries, deliberately redesigning work, micro-rituals and routines, and recovery tactics such as taking micro-breaks. Together, these strategies demonstrate how resource gains can be actively produced under virtual mobility.

Many participants implemented clear end-of-day routines or context switches to prevent work encroaching on their personal time. These practices act as cognitive barriers, stabilizing evaluations of balance and preventing rumination outside working hours as boundary settings.

Marcus described a decisive routine:

“…I sit here, facing the computer. When I'm done working, I immediately turn off the computer, turn on the TV, and get on with my life.”

Larry emphasized the role of sensory gating in achieving focus and closure, saying,

“At home, I work with my headphones on, listening to music, to shut myself off from the outside world.”

David emphasized the importance of closure cues that offices used to provide

“Even when work isn't intense, going to and from the office or doing something after work helps to close the day.”

Cognitive wellbeing was also reinforced through autonomy claims and the active redesign of tasks or job crafting. Marcus highlighted discretion:

“I have decision-making power.”

Ruby framed autonomy as authenticity and efficiency, saying,

“At this point, I worked more efficiently and in a way that felt more authentic.”

Emily connected planning discipline with ownership over outputs

“I had to act in a planned way...”

By adjusting task sequences, pacing and methods, participants achieved resource gains that translated into greater satisfaction and perceived balance.

Participants relied on subtle rituals to mark transitions and stabilize attention.

David explained that he adapted not only his attire but also his work environment to something he was used to:

“I bought a desk to work at. I worked without a desk for a month. Some work can be done lying down. Simple tasks can be done lying down, but when I need something to focus on, I needed a desk like the ones in offices....I can stay in my comfortable clothes until the meeting. Before the meeting starts, I wear things with better-looking collars. If it's a bigger meeting, I wear something with an even better collar.”

“Even at the beginning, I wore a tie and bow tie to team meetings.” (Marcus)

Larry combined spatial and sensory rituals, such as closing the door and putting on headphones, to signal a shift in mental mode. These micro-rituals acted as cognitive anchors, replacing lost office routines and supporting consistent performance standards. Another recurring strategy was the introduction of structure to counter temporal drift. Emily explained how routinization reduced procrastination:

“Then every day became standardized; the program was clear.”

Jessia captured the opposite risk and why structure matters:

“When you're at home, you learn to procrastinate.”

Ruby stressed the importance of self-discipline in remote settings

“But I was more disciplined at home.”

Time-blocking, simple rules and visible schedules helped to translate autonomy into dependable completion, thereby bolstering cognitive evaluations of control and accomplishment.

Participants also emphasized the importance of short, deliberate pauses to maintain attention and stave off fatigue as recovery. Jack's reflection is emblematic

“Short breaks... 5 to 10 min breaks are very important for maintaining concentration.”

Marcus contrasted long, unfocused lulls with intentional breaks bracketed by scheduled calls

“Because my meeting is scheduled, my break is scheduled.”

Recovery tactics helped preserve mental clarity, which participants linked to satisfaction at the end of the day and reduced cognitive overload. Participants reported using digital tools to maintain communication with colleagues and managers and to accomplish their tasks. However, excessive use of such tools can also lead to fatigue and burnout, leading to decreased life satisfaction (Rußmann et al., 2025).

### 5.4 Implications and future research

The findings have important implications for organizational practice and policy. At an organizational level, they emphasize the importance of balancing flexibility with stability. Temporal acceleration and drift emphasize the importance of temporal markers, such as start and end times, shared breaks or regular check-ins, in stabilizing affective rhythms. From a human resources perspective, it is essential to promote autonomy while preventing overload. Boundary setting and time management are heavily influenced by leadership behaviors and the strength of digital infrastructures. Training in boundary management, job crafting and recovery practices can help employees to view flexibility as a source of benefit rather than strain. Because remote work affects employees differently depending on gender, family structure, and occupation, organizational practices should be context-sensitive rather than uniform. Additionally, the erosion of collegial ties suggests that managers should foster social connection through virtual coffee breaks, or peer support groups, while modeling healthy boundary setting behaviors. Virtual community-building that enhances digital social capital can help counteract social isolation in remote settings.

At the policy level, virtual mobility should be recognized as a distinctive working condition with implications for productivity and wellbeing. Regulations could include provisions for the right to disconnect, mental health support, and structured digital work arrangements. Deregulating working hours is also highly risky for work-life balance and gender equality. For women, employer-driven flexibility can create more stress due to home-work burdens, while for men, autonomy can increase the risk of long working hours and a shift away from home responsibilities (Lott, 2020). Therefore, predictability and reliability of working hours should be maintained as organizational policy.

The study's main strengths lie in its novel conceptualization of remote work as virtual mobility, its use of digital ethnography to capture lived experiences, and its theoretical synthesis of COR and boundary theory. Limitations include the small but relatively homogeneous sample size, which restrict generalizability. Future research should employ longitudinal or diary-based qualitative designs, as well as mixed-methods approaches to examine the intersection with gender, family status, and inequality. Additionally, future studies should expand their sample size to enable comparisons across sectors, household structures, and remote work arrangements.

## 6 Conclusion

This study examined remote work as a distinctive form of virtual mobility, showing how it affects wellbeing in terms of time, boundaries and resources. Firstly, it defines remote working as a type of virtual mobility, broadening the scope of mobility research beyond physical movements (Cresswell, 2006; Ren et al., 2024). While existing scholarship has primarily examined the economic benefits of mobility (Fortunati and Taipale, 2017), this study demonstrates that mobility in the digital realm also has significant implications for SWB. Second, the study contributes to wellbeing research by foregrounding its multidimensional nature. Affective wellbeing fluctuates with the temporal rhythms of acceleration, while SWB is reshaped through the permeability of boundaries and the erosion or selective strengthening of collegial ties (Diener et al., 1999; Ryan and Deci, 2001). Cognitive wellbeing, meanwhile, hinges on strategies for regulating resources, such as setting boundaries, job crafting and ritualized coping (Helliwell et al., 2025). Thirdly, COR emphasizes that autonomy, flexibility, and control over rhythms can be perceived as gains in resources, whereas social isolation, blurred routines, and loss of collegial support can be perceived as losses in valuable resources (Hobfoll, 1989). Boundary theory illuminates how permeability and temporal drift erode the cognitive and social markers that sustain stability, requiring individuals to actively reconstruct boundaries through rituals, or selective social interactions (Ashforth et al., 2000). Finally, the study's emphasis on lived temporalities and coping practices highlights the importance of examining processes rather than outcomes. Much of the existing literature has focused on whether remote working is beneficial or detrimental to wellbeing (Ferrara et al., 2022). In contrast, this study demonstrates how employees make sense of virtual mobility in their everyday lives. Framing subjective time perception as a psychological mechanism rather than a contextual condition allows the analysis to move beyond dichotomous debates on the effects of remote work, highlighting the contingent and processual nature of wellbeing in digital work environments.

## Data Availability

The raw data supporting the conclusions of this article will be made available by the authors, without undue reservation.
